# A Point Mutation in the Hair Cell Nicotinic Cholinergic Receptor Prolongs Cochlear Inhibition and Enhances Noise Protection

**DOI:** 10.1371/journal.pbio.1000018

**Published:** 2009-01-20

**Authors:** Julian Taranda, Stéphane F Maison, Jimena A Ballestero, Eleonora Katz, Jessica Savino, Douglas E Vetter, Jim Boulter, M. Charles Liberman, Paul A Fuchs, A. Belén Elgoyhen

**Affiliations:** 1 Instituto de Investigaciones en Ingeniería Genética y Biología Molecular, Consejo Nacional de Investigaciones Científicas y Técnicas, Buenos Aires, Argentina; 2 Department of Neuroscience, Tufts University School of Medicine, Boston, Massachusetts, United States of America; 3 Department of Otology and Laryngology, Harvard Medical School and Eaton–Peabody Laboratory, Massachusetts Eye and Ear Infirmary, Boston, Massachusetts, United States of America; 4 Department of Psychiatry and Biobehavioral Sciences, Hatos Research Center for Neuropharmacology, Brain Research and Molecular Biology Institutes, University of California, Los Angeles, United States of America; 5 Department of Otolaryngology, Head and Neck Surgery, and Center for Hearing and Balance, The Johns Hopkins University School of Medicine, Baltimore, Maryland, United States of America; 6 Departamento de Farmacología, Facultad de Medicina, Universidad de Buenos Aires, Buenos Aires, Argentina; University of Texas, Austin, United States of America

## Abstract

The transduction of sound in the auditory periphery, the cochlea, is inhibited by efferent cholinergic neurons projecting from the brainstem and synapsing directly on mechanosensory hair cells. One fundamental question in auditory neuroscience is what role(s) this feedback plays in our ability to hear. In the present study, we have engineered a genetically modified mouse model in which the magnitude and duration of efferent cholinergic effects are increased, and we assess the consequences of this manipulation on cochlear function. We generated the *Chrna9L9′T* line of knockin mice with a threonine for leucine change (L9′T) at position 9′ of the second transmembrane domain of the α9 nicotinic cholinergic subunit, rendering α9-containing receptors that were hypersensitive to acetylcholine and had slower desensitization kinetics. The *Chrna9L9′T* allele produced a 3-fold prolongation of efferent synaptic currents in vitro. In vivo, *Chrna9L9′T* mice had baseline elevation of cochlear thresholds and efferent-mediated inhibition of cochlear responses was dramatically enhanced and lengthened: both effects were reversed by strychnine blockade of the α9α10 hair cell nicotinic receptor. Importantly, relative to their wild-type littermates, *Chrna9^L9′T/L9′T^* mice showed less permanent hearing loss following exposure to intense noise. Thus, a point mutation designed to alter α9α10 receptor gating has provided an animal model in which not only is efferent inhibition more powerful, but also one in which sound-induced hearing loss can be restrained, indicating the ability of efferent feedback to ameliorate sound trauma.

## Author Summary

Nicotinic cholinergic receptors are essential to higher order brain function. Structurally, these operate through a myriad of ligand-gated pentameric arrangements of different homologous subunits. Here, we report progress in understanding the structural properties of a neuronal nicotinic receptor at the synapse. Receptors assembled from two nicotinic cholinergic subunits (α9 and α10) serve exclusively at the synapse between central nervous system descending fibers and cochlear hair cells. This enabled us to show direct causality between a point mutation of the α9 subunit, and predicted alterations in the synaptic strength in sensory hair cells of the cochlea of α9 point mutant mice. Furthermore, this single mutation results in profound enhancement of central nervous system feedback to the cochlea. And finally, as a consequence, mutant mice possessing this altered receptor have substantially improved resistance to traumatic sound. Thus, central neuronal feedback on cochlear hair cells provides an opportunity to define one specific role that nicotinic receptors can play in the nervous system, enabling study from biophysical to behavioral levels and promoting a target for the prevention of noise-induced hearing loss.

## Abstract 3

A point mutation in the cochlear hair cell nicotinic cholinergic receptor leads to strengthened central nervous system feedback to the cochlea and enhances protection from noise-induced hearing loss.

## Introduction

In bringing information about the world to an individual, sensory systems perform a series of common functions. Each system responds with some specificity to a stimulus, and each one employs some specialized receptor cells at the periphery to translate specific stimuli into electrical signals that all neurons can use. That initial electrical event begins the process by which the central nervous system constructs an orderly representation of, for example, sounds, odors, tastes, and objects. Thus, basic sound detection begins when sound waves strike the eardrum, which transmits that physical stimulus to the organ of Corti within the cochlea, the sensory epithelium of the mammalian inner ear. Here, the primary receptor cells known as inner hair cells (IHCs) transform the information into electrical signals that are sent to the central nervous system by the auditory nerve [1]. However, unlike vision, touch, and the chemical senses, sound processing is modulated by efferent signals that travel in reverse, from the brain back to the inner ear [2]. One fundamental question in auditory neuroscience is what role(s) this feedback plays in our ability to hear.

**Figure 1:**
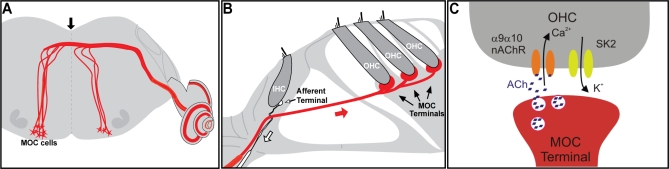
Schematics Showing the Central Origin (A) and Peripheral Projections (B) of the MOC Fibers and the Cholinergic Synapse onto OHCs in the Mature Organ of Corti (C) MOC efferent neurons are located in the superior olivary complex of the brainstem and project to the cochlea, where they make direct synaptic contacts at the base of the OHCs. At this synapse, ACh is released. It binds to α9α10 receptors present at the OHCs, leading to Ca^2+^ influx and the subsequent activation of Ca^2+^-dependent SK2 K^+^ channels and hair cell hyperpolarization. The black arrow in (A) indicates the place of electrical stimulation to activate the MOC fibers. The white arrow in (B) indicates the afferent fibers that bring the information from the IHCs to the central nervous system, and the red arrow indicates the MOC fibers. For approximately 10 d after birth (before the onset of hearing), cholinergic efferents temporarily synapse directly with IHCs (not shown in the figure) and provide a useful experimental target for the study of altered α9α10 receptors.

The medial olivocochlear (MOC) efferents (Figure 1A) originate in the medial portion of the superior olivary complex and project to outer hair cells (OHCs; Figure 1B) of the organ of Corti, where large synaptic contacts are formed [2]. In contrast to IHCs, OHCs act as biological motors to amplify the sound-evoked motions of the organ of Corti through a type of somatic electromotility generated by a specialized membrane protein known as prestin. Activation of the MOC pathway, either by sound or by shock trains delivered to the bundle at the floor of the IVth ventricle (Figure 1A), reduces cochlear sensitivity through the action of the neurotransmitter acetylcholine (ACh) on nicotinic receptors (nAChRs) at the base of OHCs (Figure 1C) [3]. Although significant progress has been made in defining the cellular mechanisms of hair cell inhibition [3], the functional role(s) of this sound-evoked feedback system, including control of the dynamic range of hearing [2], improvement of signal detection in background noise [4–6], mediating selective attention [7,8], and protection from acoustic injury [9], remain controversial.

In addition to their significance in auditory processing, efferent cholinergic synapses provide a unique opportunity to assess the function of neuronal nAChRs. In contrast to nAChR-expressing cells in the central nervous system [10], hair cells are compact and isopotential, and receive no other synaptic inputs. Moreover, compared to most other neuronal nAChRs, whose subunit composition remains challenging to elucidate [11], the oligomeric structure of the hair cell nAChR has been defined: α9 and α10 subunits arrange into a pentameric assembly with a likely (α9)_2_(α10)_3_ stoichiometry [12–15]. Current data support the notion that activation of the α9α10 nAChR leads to an increase in intracellular Ca^2+^ and the subsequent opening of small conductance Ca^2+^-activated K^+^ SK2 channels, thus leading to hyperpolarization of hair cells [3,16,17], (Figure 1C).

In the present study, we have generated a genetically modified mouse model in which the magnitude and duration of the MOC efferent effect is increased, and assessed the consequences of this manipulation on auditory thresholds and susceptibility to noise-induced hearing loss. We have substituted a threonine for a leucine at position 9′ (*L9′T*) of the second transmembrane domain of the α9 subunit and examined the neurotransmitter responses, synaptic efficiency, and cochlear function of *Chrna9^wt/wt^*, *Chrna9^wt/L9′T^*, and *Chrna9^L9′T/L9′T^* mice. We show that the L9′T mutation produced an increase in sensitivity to ACh, and decreased rates of ACh-induced desensitization. More significantly, synaptic currents were dramatically prolonged in the OHCs of *Chnra9^L9′T/L9′T^* mice. Consistent with these effects, *Chnra9^L9′T/L9′T^* mice had elevated acoustic thresholds, and shock-evoked MOC activation produced both enhanced and prolonged cochlear suppression. This enhanced efferent inhibitory drive attenuated sound-induced, permanent acoustic injury. Our results establish efferent feedback inhibition's function in preventing acoustic trauma, and provide preliminary characterization of a mouse model to explore additional physiological functions of MOC innervation. In addition, it motivates further exploration of the efferent synapse as a model system to study fast neurotransmission mediated by a neuronal nAChR.

## Results

**Figure 2:**
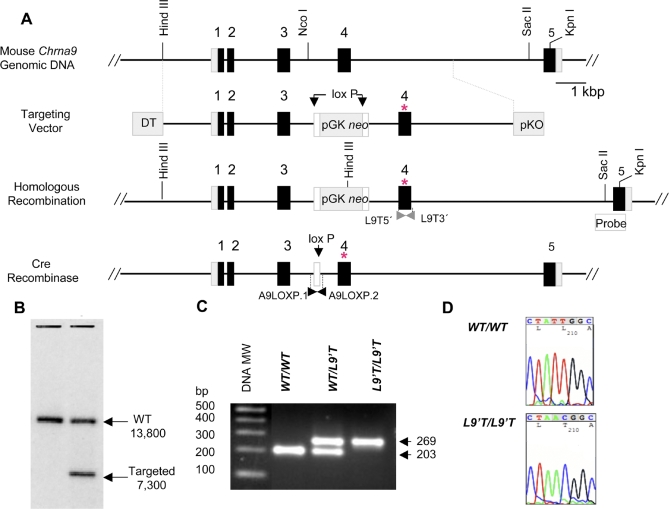
Genetic Engineering and Genotyping of *Chrna9L9'T* Mutant Mice (A) Gene structures and restriction maps for the targeting construct, wild-type, and recombinant alleles (before and after removal of the neomycin resistance cassette) are shown. Black boxes represent the exons of the *Chrna9* gene. The introduction of the mutation is shown by an asterisk (*). (B) An autoradiograph of a genomic Southern blot using DNA prepared from wild-type (left lane) and successfully targeted ES cells (right lane) and hybridized to a ^32^P-labled DNA probe prepared from the 962-bp SacII-KpnI fragment shown. Wild-type (*WT*) ES cell DNA yield a fragment of 13,800 bp, whereas ES cells that have undergone homologous recombination yield a 7,300-bp fragment. (C) Routine genotyping of *neo*-deleted *Chrna9L9′T* mutant mice performed by PCR from isolated tail biopsy tissue genomic DNA samples and amplimers flanking the *lox P* footprint (white box). A representative gel indicating the results for wild-type, heterozygous, and homozygous knockin mice is shown. The PCR fragment length for the wild-type *Chrna9* allele is 203 bp, and 269 bp for the mutant allele. MW, molecular weight. (D) A PCR fragment using amplimers flanking the *L9′T* mutation (Figure 1A) was obtained from genomic DNA isolated from tail biopsies and sequenced to check the integrity of the mutation in each breeding pair. A representative electropherogram indicating the results for wild-type and homozygous knockin mice is shown. The wild-type sequence at nucleotide position 210, corresponding to the codon at position 9′ in the second transmembrane domain, is TTG, encoding leucine; the mutant sequence is ACG, encoding threonine.

The targeting construct and generation of *Chrna9L9′T* mutant mice are described in the Materials and Methods section and Figure 2. Both *Chrna9^wt/L9′T^* and *Chrna9^L9′T/L9′T^* mutant mice were viable through adulthood, fertile, showed the expected gender and Mendelian genotype ratios, and exhibited no overt behavioral phenotype. Gross morphology of the cochlear duct was normal as seen in light microscopic evaluation of plastic sections of osmium-stained cochleae (Figure S1). As assessed by quantitative real-time PCR (RT-PCR), the expression level of cochlear nAChR α9 subunit mRNA was similar in *Chrna9^wt/wt^* and *Chrna9^L9′T/L9′T^* mice (Table S1). In addition, the expression levels of genes encoding proteins known to localize to the olivocochlear (OC) synaptic complex (for example, the nAChR α10 subunit and SK2 channel) were not changed in mutant mice (Table S1). Lastly, the electrophysiology of IHCs and OHCs was normal, as indicated by similar voltage-dependent K^+^ currents (Figure S2). Hair cell membrane area measured by capacitance was identical across phenotypes (*Chrna9^wt/wt^*: 8.3 ± 0.2 pF, *n* = 19 animals, 41 IHCs; *Chrna9^wt/L9′T^*: 8.4 ± 0.1 pF; *n* = 14 animals, 41 IHCs; *Chrna9^ L9′T /L9′T^*: 8.6 ± 0.1 pF, *n* = 26 animals, 79 IHCs, measured at P9–P11; *Chrna9^wt/wt^*: 8.0 ± 0.4 pF, *n* = 5 animals, 13 OHCs; and *Chrna9^ L9′T /L9′T^*: 8.1 ± 0.2 pF, *n* = 8 animals, 19 OHCs, measured at P10–P11).

### Hypersensitive and Slowly Desensitizing nAChRs in Mutant Mice

In *Xenopus* oocytes, α9α10 nAChRs assembled from mutant α9L9′T subunits exhibit an increased sensitivity to ACh, a reduced desensitization rate, and an increased channel mean open time [18]. To determine whether similar changes occurred in hair cells of L9′T mutant mice, we examined the response of neonatal IHCs to exogenous ACh. IHC nAChRs, which are expressed transiently before the onset of hearing, are of the α9α10 subtype and are functionally indistinguishable from those that arise later in OHCs [19,20]. Since recordings are more frequently and reliably obtained from neonatal IHCs than OHCs, the former provided the bulk of the data presented. As shown below, a subset of our findings has been repeated in OHCs of *Chrna9L9′T* mutant mice.

**Figure 3:**
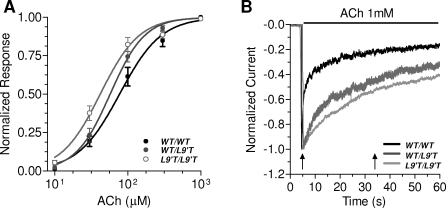
Hypersensitive and Slowly Desensitizing ACh Responses (A) Concentration-response curves to ACh performed in P9–P10 IHCs. Peak current values were normalized and referred to the maximal peak response to ACh in each case. The mean and S.E.M. of experiments performed in *n* = 7 cells (4 animals) for *Chrna9^wt/wt^*, 9 cells (4 animals) for *Chrna9^wt/L9′T^*, and 7 cells (4 animals) for *Chrna9^L9′T/L9′T^* are shown; (B) Wave-form of responses to ACh in P6–P10 IHCs voltage-clamped at −90 mV. Representative responses to the application of 1 mM ACh during 1 min are shown. *WT*, wild type.

As observed in Figure 3A, IHCs of *Chrna9^wt/L9′T^* and *Chrna9^L9′T/L9′T^* mice responded to ACh at P6–P10. In each case, ACh dose-response curves of IHCs were left-shifted when compared to wild type, with a concomitant decrease in the half maximal effective concentration (EC_50_). The EC_50_ was significantly different (*p* < 0.01) between homozygous mutant and wild-type IHCs (*Chrna9^wt/wt^*: EC_50_, 90.8 ± 18.2 μM, Hill coefficient 1.6 ± 0.1, *n* = 4 animals, 7 cells; *Chrna9^wt/L9′T^*: EC_50_, 57.2 ± 6.5 μM, Hill coefficient 2.2 ± 0.1, *n* = 4 animals, 9 cells; and *Chrna9^ L9′T /L9′T^*: EC_50_, 41.7 ± 5.7 μM, Hill coefficient 2.1 ± 0.2, *n* = 4 animals, 7 cells). During prolonged (>60 s) application of 1 mM ACh at −90 mV (Figure 3B), the current was entirely inward since the ACh response was recorded in isolation from the SK2 component (see below). The evoked membrane current in wild-type IHCs decayed with a complex time course. In IHCs of both *Chrna9^wt/L9′T^* and *Chrna9^L9′T/L9′T^* mice, ACh-evoked membrane current was more sustained than that seen in wild-type mice (i.e., showed reduced desensitization, as previously shown with recombinant α9α10 nAChRs expressed in oocytes [18]). The fraction of current remaining after a 30-s ACh application was 0.29 ± 0.04 in *Chrna9^wt/wt^* (*n* = 10 animals, 15 cells), 0.45 ± 0.05 in *Chrna9^wt/L9′T^* (*n* = 9 animals, 12 cells, *p* < 0.05), and 0.48 ± 0.06 in *Chrna9^ L9′T/L9′T^* (*n* = 6 animals, 10 cells, *p* < 0.05).

### Prolonged Synaptic Currents in Mutant IHCs

**Figure 4:**
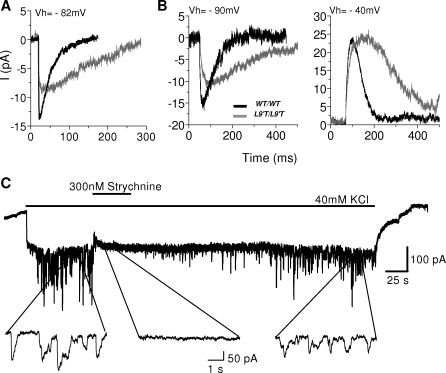
Prolonged Synaptic Currents in IHCs Are Blocked by Strychnine (A) Superimposed representative traces from individual cells of nAChR-only sIPSCs in IHCs from a *Chrna9^wt/wt;^* (27 events) and a *Chrna9^L9′T/L9′T^* (27 events) mice at a *V*_hold_ (Vh) of −82 mV. (B) Superimposed representative traces from individual cells of nAChR+SK synaptic currents in IHCs at a *V*_hold_ of −90 mV from a *Chrna9^wt/wt^* (84 events) and a *Chrna9^L9′T/L9′^* (16 events), and of −40 mV from a *Chrna9^wt/wt^* (54 events) and a *Chrna9^L9′T/L9′^* (14 events). The representative traces shown are the average of the different events in one cell (number of cells per experiment indicated in Table 1). *WT*, wild type. (C) Representative traces (*n* = 4 IHCs, 2 animals) of synaptic currents evoked by 40 mM external K^+^ at *V*_hold_ of −90 mV in *Chrna9^L9′T/L9′^*. The blow-ups show IPSCs, which disappear in the presence of 300 nM strychnine.

Following efferent transmitter release in vitro, spontaneous inhibitory postsynaptic currents (sIPSCs) can be measured in neonatal IHCs. As determined for MOC synapses located on OHCs, the IHC inhibitory postsynaptic currents (IPSCs) result from ion flux through α9α10 nAChRs and calcium-activated SK2 channels [19,20], (Figure 1C). To only measure currents through α9α10 nAChRs, responses were recorded using pipettes filled with KCl-BAPTA (thereby preventing calcium from activating SK2 channels) and 5 nM apamin (an SK channel blocker) in the extracellular solution. As shown in Figure 4A and detailed in Table 1, these “nAChR-only” sIPSCs of mutant mice were dramatically prolonged compared to wild type. There was a 6-fold increase in the *τ*_decay_, no difference in *τ*_rise_, and a 3-fold increase in the duration at half amplitude (halfwidth). In addition, the sIPSCs in *Chrna9^L9′T/L9′T^* IHCs had significantly smaller amplitudes than those of wild-type IHCs. Despite the amplitude reduction, overall charge transfer (as indicated by the area value) of the “nAChR-only” synaptic current was more than doubled in *Chrna9^L9′T/L9′T^* when compared to *Chrna9^wt/wt^* IHCs.

**Table 1:**
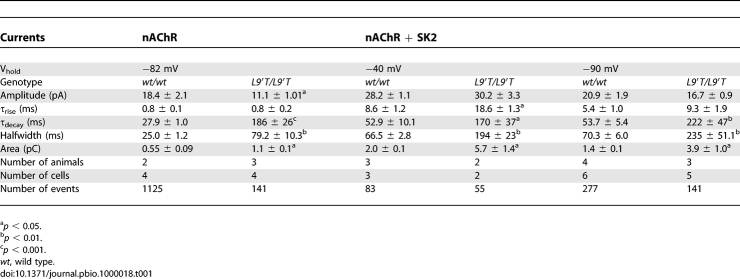
Synaptic Currents in IHCs from *Chrna9^wt/wt^* and *Chrna9^L9′T/L9′T^* Mice

When sIPSCs were recorded with KCl-EGTA in the pipette, currents were inward at −90 mV and outward at −40 mV (Figure 4B). As noted for wild-type IHCs ([19,20] and present results), the polarity shift of the sIPSC indicates that SK2 channel gating occurs in the *Chrna9L9′T* mutant mice. At −90 mV, sIPSCs of *Chrna9^L9′T/L9′T^* mice were dramatically prolonged compared to *Chrna9^wt/wt^*, with a 4- and 3-fold increase in the *τ*_decay_ and halfwidth, respectively. At −40 mV, *τ*_decay_ and halfwidth were 3-fold greater than in wild type, and a slight increase in *τ*_rise_ also was observed. The peak current at −40 mV, carried by SK2 channels, was equivalent in *Chrna9^wt/wt^* and *Chrna9^L9′T/L9′T^* IHCs. Nonetheless, the smaller amplitude nAChR-only current observed in IHCs of mutant mice activates SK2 channels as effectively as in wild-type IHCs. Indeed, the area under the *Chrna9^L9′T/L9′T^* SK2 current was nearly three times greater than that of the *Chrna9^wt/wt^* SK2 current. Presumably, this enhancement results from the increased total calcium ion flux during the prolonged activity of the nAChRs in mutant mice.

As shown in Figure 4C, synaptic activity evoked by 40 mM K^+^ in IHCs was reversibly blocked by the α9α10 blocker strychnine (300 nM) [13], indicating that the synaptic activity observed in *Chrna9^L9′T/L9′T^* was in fact mediated through mutant α9α10 nAChRs.

### Prolonged Synaptic Currents and Coupling to SK2 Channels in Mutant OHCs

**Figure 5:**
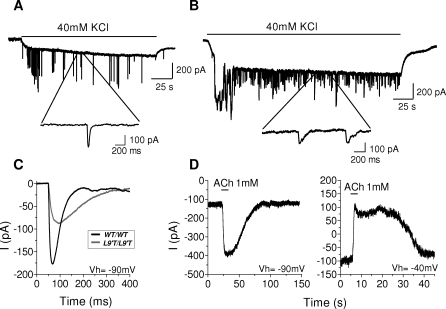
Prolonged Synaptic Currents in OHCs (A) Representative traces of IPSCs evoked by 40 mM K^+^ in OHCs of P10–P11 *Chrna9^wt/wt^* mice at a *V*_hold_ of −90 mV. The insets show synaptic currents on an expanded time scale. (B) Same as in (A) but for *Chrna9^L9′T/L9′T^* mice. (C) Superimposed representative traces of nAChR+SK2 synaptic currents from a *Chrna9^wt/wt;^* (39 events) and a *Chrna9^L9′T/L9′T^* (77 events) mice. The representative traces shown are the average of the different events in one cell (number of cells per experiment indicated in Table 2). Vh, *V*_hold_; *WT*, wild type. (D) Representative responses of OHCs to 1 mM ACh at a V_hold_ of −90 mV and −40 mV.

Since hair cell sIPSCs were infrequently observed, synaptic currents and the effects of exogenous ACh were measured in P10–P11 OHCs using high-potassium buffer (40 mM K^+^ plus EGTA, minus apamin) to depolarize the efferent terminals (Figure 5A and 5B). Under these recording conditions, the synaptic waveform includes current through both the nAChR and associated SK2 channels (entirely inward at −90 mV since 40 mM K^+^ shifts the equilibrium potential of this cation to −33 mV). Analysis of waveforms revealed a 4- and 3.5-fold increase in *τ*_decay_ and halfwidth, respectively, and a 0.6-fold reduction in amplitude (Figure 5C and Table 2) in *Chrna9^L9′T/L9′T^* IPSCs compared to *Chrna9^wt/wt^*, much as was observed in IHCs. Despite a drop in amplitude, the overall charge transfer (as indicated by the area value) was 2-fold larger in *Chrna9^L9′T/L9′T^* compared to *Chrna9^wt/wt^* OHCs.

**Table 2:**
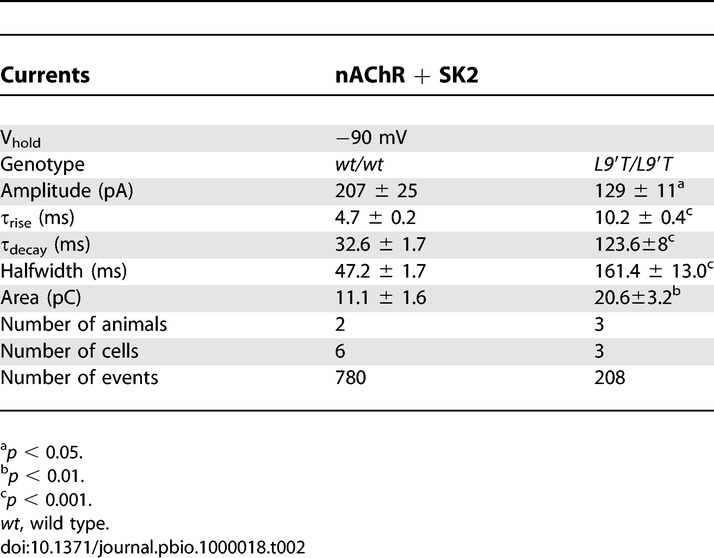
Synaptic Currents in OHCs from *Chrna9^wt/wt^* and *Chrna9^L9′T/L9′T^* Mice

Under these recording conditions, synaptic currents reflect ion flux through both nAChRs and associated SK2 channels. The presence of an ACh-evoked SK2 current was demonstrated directly by application of ACh to *Chrna9^L9′T/L9′T^* OHCs held at different membrane potentials. As shown in Figure 5D, ACh responses were outward at −40 mV, confirming the activation of potassium currents in mutant OHCs.

### Increased Cochlear Thresholds in Mutants Can Be Reversed with Strychnine

We examined cochlear responses in *Chrna9L9′T* mutant mice via auditory brainstem responses (ABRs) and distortion product otoacoustic emissions (DPOAEs). Wave 1 of the ABR, which is recorded from scalp electrodes in response to short tone pips, represents the summed activity of cochlear nerve fibers projecting from IHCs (Figure 1B) to the cochlear nucleus, the first central nucleus in the ascending auditory pathway [21]. The DPOAEs are sounds created within the cochlea, amplified by the action of OHCs and propagated through the middle ear back to the ear canal, where they can be measured with a microphone [22]. Given that the generation of DPOAEs requires neither IHCs nor cochlear nerve fibers [23], a comparison of ABRs and DPOAEs can provide insight into the locus of any dysfunction.

**Figure 6:**
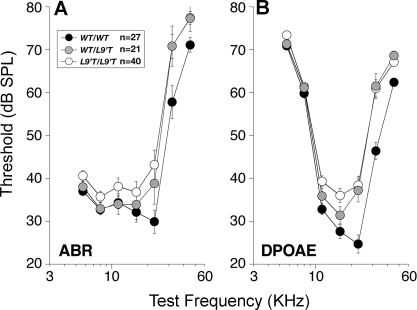
Baseline Cochlear Thresholds Are Elevated in Mutant Ears, as Measured by either ABRs (A) or DPOAEs (B) Mean group values ± S.E.M. are shown and numbers of ears measured in each group are indicated in the key in (A). ABR thresholds were identified by visual inspection of the stacked level-series waveforms. DPOAE threshold was defined by interpolation as the sound pressure of the f_2_ primary required to evoke a DPOAE of 0 dB SPL. *WT*, wild type.

As shown in Figure 6A, mean ABR thresholds were elevated by 5–15 dB in both *Chrna9^wt/L9′T^* (*F*_(1,55)_ = 4.30, *p* = 0.043) and *Chrna9^L9′T/L9′T^* (*F*_(1,79)_ = 7.87, *p* = 0.06) when compared to *Chrna9^wt/wt^* mice. DPOAE thresholds (Figure 6B) were also elevated by 5–15 dB in both *Chrna9^wt/L9′T^* (*F*_(1,53)_ = 14.82, *p* < 0.001) and *Chrna9^L9′T/L9′T^* (*F*_(1,77)_ = 9.20, *p* = 0.003). Threshold shifts were larger at frequencies above 16 kHz and were similar in magnitude whether measured by ABRs or DPOAEs, suggesting that all dysfunction can be explained by changes in OHC contributions to cochlear amplification.

**Figure 7:**
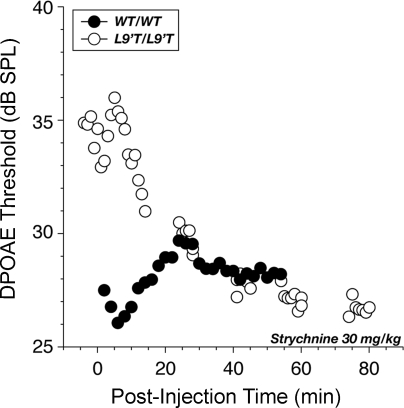
Strychnine Improves Cochlear Thresholds in Mutant Mice By 1 h postinjection, thresholds in mutant mice recovered to the mean value from wild types (23 dB SPL at 22.6 kHz: Figure 5B). Strychnine produced no systematic effects on wild-type (*WT*) thresholds. Here, DPOAE thresholds for f_2_ = 22.6 kHz were repeatedly measured before and after a 30-mg/kg i.p. strychnine injection in a *Chrna9^wt/wt^* and a *Chrna9^L9′T/L9′T^*. The effect of strychnine was concentration-dependent as shown in Figure S3.

Since MOC activity in vivo decreases OHC-based amplification of cochlear responses [2], we asked whether the increased cochlear thresholds might arise from enhanced synaptic currents through mutant α9-containing channels in response to the normal low level of spontaneous MOC activity [24]. To test this hypothesis, we injected mice with strychnine (30 mg/kg, intraperitoneally [i.p.]), a potent blocker of α9α10 channels [13,25]. Indeed, as shown in Figure 7, strychnine improved acoustic thresholds in *Chrna9^L9′T/L9′T^* mice by almost 9 dB, thus restoring the baseline thresholds to that seen in the wild type.

### Shock-Evoked OC Suppression Is Slowed, Enhanced, and Prolonged in Mutants

**Figure 8:**
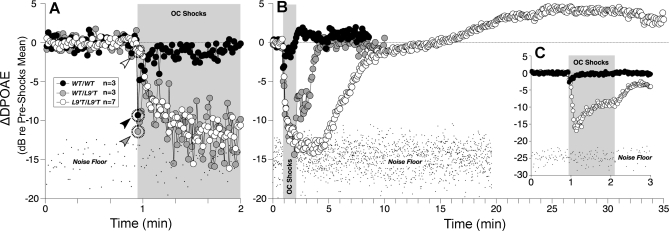
OC-Mediated Suppression of Cochlear DPOAEs Is Slowed, Enhanced and Prolonged in Mutant Mice (A) and (B): DPOAE amplitudes measured before, during, and after a 70-s shock train to the OC bundle (grey boxes) are shown on two different time scales to emphasize the onset effects (A) and the offset effects (B) of OC activation. DPOAE amplitudes from each experiment are normalized to the average preshock value and then averaged across ears within a genotype (group sizes are shown in the key in (A). Arrowheads in (A) indicate the first point after shock-train onset for each genotype: data acquisition times are approximately 1s/point. For (A) and (B), the f_2_ primary was at 22.6 kHz, and primary levels were adjusted to produce a DPOAE approximately 15 dB above the noise floor: suppression is so strong in mutant ears that the DPOAEs are driven into the noise. *WT*, wild type. (C) To reveal full suppression magnitude, we raised primary levels until preshock DPOAEs were 25 dB above the noise floor: peak suppression in *Chrna9^L9′T/L9′T^* reached approximately 17 dB, whereas for *Chrna9^wt/wt^*, effects were less than approximately 5 dB at these higher stimulus levels.

Activation of MOC efferents normally decreases the OHC contribution to cochlear amplification. Thus, the electrical stimulation of the MOC fibers at the floor of the IVth ventricle (Figure 1A, black arrow) results in a decrease in the amplitude of the DPOAEs [25]. To assess MOC function in vivo, DPOAEs were measured before, during, and after a 70-s train of shocks to the olivocochlear bundle. In wild-type mice, DPOAE suppression is seen immediately after shock-train onset (Figure 8A, black arrowhead) and then adapts to a steady state, which is maintained throughout the shock epoch (Figure 8A and 8B). In *Chrna9L9′T* mutant mice, suppression had a much slower onset (Figure 8A, white arrowhead), but continued to grow during the shock train (Figure 8A and 8B) until responses disappeared into the noise floor. To determine the full suppression magnitude, we raised the level of the acoustic stimulus (Figure 8C): peak suppression in *Chrna9^L9′T/L9′T^* reached approximately 17 dB, whereas for *Chrna9^wt/wt^*, the maximum suppression was less than approximately 5 dB. In homozygous mutants, suppression persisted for almost 10 min after the end of the shock train, with a slow recovery to baseline (Figure 8B). Moreover, there is a prominent “overshoot,” which, except for a slower time course, is reminiscent of the post-shocks enhancement seen in wild-type mice [25]. As shown in Figure 8A and 8B, responses in *Chrna9^wt/L9′T^* were intermediate between those of homozygous mutant and wild-type mice.

**Figure 9:**
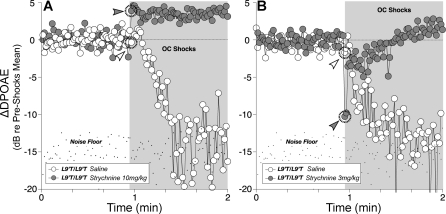
Strychnine Blocks OC Suppression in Mutant Ears (A) At 10 mg/kg, strychnine injection blocked OC-mediated DPOAE suppression in mutant mice, leaving only the slow OC-mediated enhancement seen in *Chrna*9 null mice [25]. (B) At a lower strychnine dose (3 mg/kg), the onset of suppression in the mutant mice becomes more rapid, closely resembling the wild-type response without strychnine (Figure 7). Arrowheads in (A) and (B) indicate the first point after shock-train onset for each genotype: data acquisition times are approximately 1/point.

As with wild-type mice [25], suppression could be blocked by strychnine (10 mg/kg), leaving only the postshock enhancement (Figure 9A, grey arrowhead), suggesting that the increased suppression in *Chrna9L9′T* mutants was due to the enhanced activity of α9α10 receptors. With a lower dose of strychnine (3 mg/kg), suppression in *Chrna9^L9′T/L9′T^* resembled the normal *Chrna9^wt/wt^* response (i.e., reduced suppression magnitude but with rapid onset and decay; Figure 9B, grey arrowhead).

### *Chrna9L9′T* Mutant Mice Are More Resistant to Permanent Acoustic Injury

**Figure 10:**
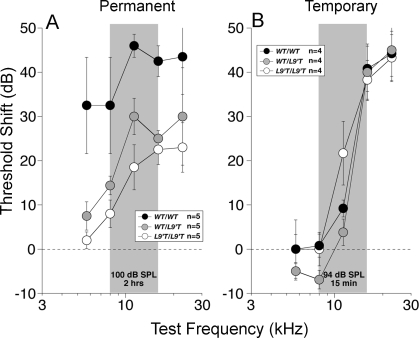
Mutant Mice Are More Resistant to Permanent Acoustic Injury (A) Permanent noise-induced threshold shift measured 1 wk after exposure to an 8–16-kHz noise band at 100 dB for 2 h. (B) Temporary noise-induced threshold shifts measured 6 h after exposure to the same noise band at 94 dB for 15 min. Each panel shows mean thresholds ± S.E.M. for the group, with group sizes indicated in the symbol key. *WT*, wild type.

Given that MOC activation most likely results in a decrease in acoustic injury [26–28], we investigated the susceptibility of *Chrna9L9′T* mutant mice to intense noise. Since the mechanisms underlying reversible and irreversible noise-induced threshold shifts are different [29,30], we used two different noise exposures to induce either a temporary (TTS) or permanent (PTS) threshold shift. Homozygous mutant mice showed increased resistance to permanent acoustic injury when compared to wild type (Figure 10A, 5.0–45.2 kHz, *Chrna9^L9′T/L9′T^*: *F*_(1,8)_ = 8.894, *p* = 0.018; *Chrna9^wt/L9′T^*: *F*_(1,7)_ = 6.263, *p* = 0.041). With a lower intensity exposure designed to produce a TTS, there was no significant difference in vulnerability between wild-type and mutant mice, when examined 12 h after exposure (Figure 10B).

## Discussion

The present study demonstrates that substitution of a single amino acid in transmembrane region 2 of the α9 subunit (*L9′T*) enhances nAChR function in cochlear hair cells (i.e., decreased ACh EC_50_ and slowed receptor desensitization rate), increases synaptic efficacy, and dramatically strengthens cochlear suppression in vivo. More importantly, it shows that the MOC efferent system inhibits cochlear sensitivity and protects the inner ear from acoustic injury. Thus, the efferent pathway provides a promising target for pharmacological prevention of inner ear pathologies derived from acoustic injury, such as hearing loss and tinnitus.

### The Hair Cell as a Model System to Study the Nicotinic Synapse

Understanding neurotransmission mediated by neuronal nicotinic receptors is a fundamental challenge in neuroscience, since decline, disruption, or alteration of nicotinic cholinergic mechanisms in the central nervous system contributes to dysfunctions such as epilepsy, schizophrenia, Parkinson's disease, autism, dementia with Lewy bodies, Alzheimer's disease, and addiction [10]. Although fast, direct nicotinic synaptic activity drives neurotransmission in autonomic ganglia, only rare cases of fast nicotinic transmission mediated by neuronal nAChRs have been reported in the mammalian brain. Because cholinergic neurons in the brain are usually loosely distributed and often sparsely innervate broad areas, it is experimentally difficult to stimulate a large number of those neurons and to record selectively from their postsynaptic targets. Indeed, it is likely that authentic fast nicotinic transmission is present at low densities in more neuronal areas than the few that have been reported [10]. Furthermore, the variable transcription of genes coding for nAChR subunits and the possible combinatorial assembly of these subunits produces a wide structural diversity of receptor types. This imposes additional challenges for studying native synaptic neuronal nAChRs [31].

Thus, the cholinergic synapse between MOC terminals and cochlear hair cells provides a valuable model for the study of fast neurotransmission mediated by nAChRs of known composition [12,13]. Targeted mutations can be introduced and the phenotypic consequences can be analyzed at the synaptic, whole-organ, and systems level. When analyzed at the level of nAChR function, the α9 *L9′T* mutant mice reproduce what has been previously described for the recombinant receptor expressed in oocytes, i.e., a decrease in EC_50_ for ACh and a reduced desensitization kinetics [18]. Both these effects probably derive from the fact that the L9′ position of nAChRs is critical for channel gating [32] and that hydrophilic substitutions at this position lead to increased mean open times [18,33]. Moreover, these changes in channel gating properties translate into increased synaptic efficacy, as seen from the prolonged synaptic currents observed in IHCs and OHCs of mutant mice. In addition, the inhibitory signature of the efferent synapse is conserved, since the nAChR currents remain coupled to the associated SK2 currents, which were also substantially prolonged. In fact, the increase in overall charge transfer during sIPSCs between wild-type and knockin mice was more pronounced when the secondary SK2 currents were measured, than for the nAChR-only currents (Table 1), pointing to an additional amplified step, possibly calcium-induced calcium release from the nearby synaptic cistern [34]. Finally, the finding that the *L9′T* mutation does not lead to hair cell death, is distinct from effects of similar mutations in α4 and α7 nAChR subunits, which lead to death of substantia nigra dopaminergic neurons [35] and apoptotic cell death throughout the somatosensory cortex [36], respectively, most likely due to Ca^2+^ excitotoxicity. The observation that α9α10 receptors are indeed highly permeable to Ca^2+^ [37,38] points toward an efficient Ca^2+^ buffering system in hair cells. In fact, proteinaceous calcium buffers (e.g., parvalbumin-β) are expressed in OHCs at high levels, similar to those found in skeletal muscle [39].

### Functional Consequences of Enhanced nAChR Gating

The main peripheral effect of the MOC activity is to inhibit cochlear responses by decreasing the gain of the cochlear amplifier [2]. MOC neurons comprise the effector arm of a sound-evoked negative feedback system that, in a quiet environment, normally has little effect on cochlear sensitivity, since MOC neurons have low levels of spontaneous activity and do not respond to sound until levels exceed threshold by 15–20 dB [24]. The fact that the baseline threshold elevation in *L9′T* mutants can be “rescued” via strychnine, the potent α9α10 nAChR blocker, suggests that it arises from an enhancement of cholinergic effects on OHCs. Therefore, compared to *Chrna9* and *Chrna10* knockouts, in which baseline cochlear thresholds were normal [40,41], the present knockin strategy reveals cholinergic MOC effects under resting conditions. This baseline inhibition could arise from an increased sensitivity of mutant receptors to normal low levels of spontaneous ACh release from MOC terminals or to the increased probability of spontaneous channel openings of *L9′T* mutant receptors in the absence of ACh [18]. The appearance of spontaneous channel openings, as described for the mutant receptors [18], may also explain how low-dose strychnine can speed the onset kinetics of the in vivo response in mutant mice (Figure 9B), i.e., by restoring mutant channels to the closed state, from which rapid ACh-mediated channel openings can occur.

Hair cell recordings from the *L9′T* mutant mice in the present study show (1) ACh-evoked currents with a greatly reduced desensitization rate, and (2) spontaneous miniature synaptic currents with slower activation and decay kinetics. These changes in ACh response kinetics provide likely explanations for some aspects of the electrically evoked suppression of DPOAEs. The doubling of onset time for the ACh-evoked SK2 current in mutant hair cells (Table 1) must contribute to the slowed onset of MOC-mediated suppression in vivo, by reducing linear summation of postsynaptic effects. However, the slowing of suppression onset is so dramatic (<1 s in wild type vs. >20 s in mutant mice) that other possible factors, e.g., alterations of release probability or slowed facilitation in the efferent terminals [42], might also contribute to this delayed time course.

In addition to rising more slowly, MOC-mediated cochlear suppression was also larger and longer lasting in *L9′T* mutant mice. The normal adaptation of suppression during continuous MOC activation was absent in the presence of slowly desensitizing α9L9′T mutant receptors, consistent with the idea that receptor desensitization is a key factor in the decay of the wild-type response after shock-train onset. However, the dramatic prolongation of cochlear suppression after shock-train offset (500 s in mutants vs. <5 s in wild types) is too large to be explained only by the 2- to 3-fold prolongation of synaptic currents observed in the mutant hair cells. Another contributing factor may be the observation that choline, the metabolite produced when acetylcholinesterase degrades ACh released at the synapse, is a full agonist of the mutant α9α10 receptor, and only a weak partial agonist of the wild-type receptor [18]. Thus, in vivo, the suppression can only decay after choline is taken up by the synaptic terminal or otherwise diffuses away. Treatment with a low dose of the α9α10 antagonist strychnine (3 mg/kg) supports this interpretation. This treatment completely abolished the prolonged postshock suppression in mutant mice (and unmasked a fast onset). Since choline has 4-fold lower affinity for α9L9′T mutant receptors than ACh [18], a low concentration of strychnine may be differentiating the effects of the two agonists (i.e., blocking the effects of the agonist with a lower potency, choline, but leaving unchanged the effects of the high-affinity agonist, ACh), as well as modulating the resting level of desensitization. Beyond the possibility of metabolite activation, extended inhibitory effects could involve more than changes in membrane conductance. For example, it has been shown that exposure to ACh alters the stiffness and motility of OHCs isolated from the gerbil cochlea over tens of minutes [43]. Stiffness and motility of OHCs depends, at least in part, on the motor protein prestin, a molecule that has selectively evolved in mammals to subserve somatic electromotility and amplification [44,45]. Thus, it remains to be determined whether prolonged Ca^2+^ influx through mutated α9α10 receptors leads to changes in prestin structure or function resulting in prolonged MOC efferent effects.

The presence of mutant α9L9′T receptors, designed to increase the magnitude of MOC effects on OHCs, also increased the protective action of the MOC system in vivo. This is consistent with previous work showing that overexpression of wild-type α9 channels, which more modestly increased the magnitude of MOC-mediated DPOAE suppression, also increased the resistance of the ear to acoustic injury [26]. Prior in vivo studies of electrically evoked MOC activity have described both fast and slow effects of ACh on cochlear neural responses [46,47] and cochlear mechanical vibrations [48]. The fast suppression, with an onset time course of approximately 100 ms, arises from the increased K^+^ conductance in neighboring SK2 channels and the effects of the resultant OHC hyperpolarization on the magnitude of electromotility and thus on cochlear vibration amplitude. The slow suppression, with an onset time course of approximately 10 s, may require a wave of calcium-induced calcium release along the OHC membrane and appears to reflect a change in OHC stiffness [48] that also reduces cochlear vibrations. The observation that overexpression of SK2 channels does not increase resistance to acoustic injury [49], although as with α9 overexpression, it also increases the magnitude of MOC-mediated DPOAE suppression, suggests that it is the slow effects of ACh that are responsible for its protective action, rather than the hyperpolarization-induced decrease in electromotility.

There are two fundamentally different ways in which slow effects of α9 activation could reduce acoustic injury: (1) by reducing mechanical vibration of the sensory epithelium, or (2) via intracellular modifications of OHCs arising as other downstream effects of the calcium entry through α9α10 receptors. The further observation that in the α9L9′T mutants, protection was seen only for exposures intense enough to produce irreversible damage and permanent threshold shifts, and not in less traumatic exposures producing only reversible changes, argues against a general reduction of vibration amplitude as the underlying mechanism. Most of the permanent threshold elevation underlying this type of noise exposure arises from damage to the hair cell stereocilia bundles, including disarray loss and/or fusion of these modified microvilli [50], which house the mechanoelectric transduction channels. Further insight into the mechanisms linking calcium entry through nAChRs and protection from acoustic injury will require a clearer delineation of the molecular events leading up to this type of noise-induced stereocilia damage.

### Conclusions

This work shows that a point mutation in the hair cell's nAChR produces dramatic prolongation of efferent MOC inhibitory effects at both cellular and systems levels. This alteration provides enhanced protection from permanent acoustic trauma, indicating that cholinergic synaptic feedback is not only necessary, but sufficient for this effect. In addition, the enhanced activity of the modified nAChR revealed a novel tonic inhibitory effect, raising baseline acoustic thresholds over those of wild-type littermates, confirming the inhibitory nature of the MOC efferent system. Thus, this α9L9′T knockin presents new insights into the cellular mechanisms of cholinergic inhibition, as well as a promising model in which to probe the functional role of MOC efferents. In addition, the hair cell's efferent synapse, much like the neuromuscular junction, can provide insights into cholinergic signaling, and promises to be an equally informative model for studying activity-dependent synaptic function.

## Materials and Methods

### Genetic engineering and genotyping of *Chrna9L9′T* knockin mutant mice.

A HindIII-NotI (the latter derived from the construction of the library) restriction endonuclease fragment of approximately 9,500 bp encoding *Chrna9* exons 1–4 (see GenBank accession number NT_039305.7 and Figure 2A) was obtained from a mouse strain 129S4/SvJae genomic DNA library (kindly provided by Dr Bernhard Bettler, University of Basel) and subcloned into the vector pKO-Select DT (Lexicon Genetics). A 2-kbp neomycin resistance cassette flanked by two loxP sites (loxP-neo-loxP) was inserted in the NcoI restriction site within the intron located between exons 3 and 4 and the *Chrna9L9′T* mutation (*) introduced via site-directed mutagenesis using the QuickChange Site-Directed Mutagenesis kit (Stratagene) and amplimers A9sense (5′-CTCTGGGAGTGACCATCCTAacGGCCATGACTGTATTTCAGC-3′) and A9antisense (5′-GCTGAAATACAGTCATGGCCgtTAGGATGGTCACTCCCAGAG-3′). This targeting vector was used to electroporate 129S4/SvJae embryonic stem (ES) cells, and homologous recombinants were obtained following gentamicin (G418) selection and Southern blot hybridization analyses. Genomic DNA was purified from G418-resistant ES cell clones, digested with HindIII and KpnI, electrophoresed on 0.8% agarose, and hybridized to a ^32^P-labled DNA 962-bp SacII-KpnI fragment probe prepared from the DNA fragment shown in Figure 2A. Based on the sequence of the mouse *Chrna9* subunit gene (see GenBank accession number NT_039305.7), wild-type ES cell DNA yielded a fragment of 13,800 bp, whereas ES cells that have undergone homologous recombination yielded a 7,300-bp fragment (see Figure 2B). Transfection of the linearized targeting vector into murine ES cells resulted in the insertion of the *L9′T* mutation into the α9 nAChR subunit. The frequency of homologous recombination events in ES cells was 42%. Six independent ES cell lines carrying the mutation were injected into blastocysts to generate germline chimeric males and were then implanted into pseudopregnant females. Chimeric male progeny mice were backcrossed to strain C57BL/6J females, and agouti coat color was used to assess the germline status of the targeted allele. Founder males were backcrossed to wild-type 129S4/SvJae females and heterozygous N1 females mated to *cre*-expressing transgenic males (FVB/N-Tg(EIIa-cre)C5379Lmgd/J, stock 003314; Jackson Laboratory) to remove the “floxed” neomycin resistance cassette. The excision of the neo cassette and subsequent segregation and loss of the *cre* transgene were monitored via PCR. The single *loxP* site footprint and flanking regions of the targeted allele in *neo*-deleted mice were confirmed by DNA sequencing.

The *Chrna9L9′T* mutant allele has been maintained in congenic FVB.129P2-*Pde6b+ Tyrc-ch*/AntJ (stock number 004828; Jackson Laboratory) strain. C57BL/6J mice develop a marked and progressive late-onset hearing loss characterized by cochlear degeneration. The FVB background lacks this hearing loss. Moreover, the wild-type *Pde6b* allele avoids blindness due to retinal degeneration typical of the FBV strain. All experiments reported in this paper were performed using *neo*-deleted *Chrna9^wt/wt^*, *Chrna9^wt/L9′T^*, or *Chrna9^L9′T/L9′T^* mutant mice backcrossed with congenic FVB.129P2-*Pde6b+ Tyrc-ch*/AntJ stock for four to five generations (i.e., N4–N5).

Routine genotyping of *Chrna9* mice was performed using tail biopsy tissue DNA samples (Wizard Genomic DNA Purification kit; Promega), amplimers A9LOXP.1 (5′-TAC TGG CTA TCC TCC AGA CAG AGC-3′) and A9LOXP.2 (5′-AGG AGC GAG CAG AGG TCC TAA ATT-3′) (see Figure 2A), and Failsafe PCR System Kit with buffer D (Epicentre) as described by the manufacturer. PCR cycle parameters were: 95 °C, 0.5 min; 55 °C, 1.0 min; and 72 °C, 2 min for a total of 35 cycles. Reaction products were electrophoresed on 1.5% agarose, stained with ethidium bromide, and photographed. The PCR fragment length for the wild-type *Chrna9* allele is 203 bp and 269 bp for the mutant allele (see Figure 2C). In each breeding pair, the mutant status of the *Chrna9L9′′T* was assessed by sequencing of the PCR fragment obtained with amplimers L9T5′ (5′-CTCTCTGACTTCATTGAAGACG-3′) and L9T3′ (5′-CCGCACACATACAGGGTTCGAT-3′) (Figure 2D).

### Electrophysiological recordings from cochlear hair cells.

Mice were sacrificed by decapitation. All experimental protocols were carried out in accordance with the American Veterinary Medical Associations' *AVMA Guidelines on Euthanasia* (June 2007). Apical turns of the organ of Corti were excised from mice at postnatal ages P6–P11 for IHCs and P10–P11 for OHCs, and used within 3 h. Day of birth was considered postnatal day 0 (P0). Cochlear preparations were mounted under a Leica DMLFS microscope (Leica Microsystems) and viewed with differential interference contrast (DIC) using a 40× water immersion objective and a Hamamatsu C7500–50 camera with contrast enhancement (Hamamatsu). Methods to record from IHCs and OHCs were essentially as described previously [17,19].

Briefly, hair cells were identified visually with the 40× objective and during recordings, by the size of their capacitance (7 to 12 pF), by their characteristic voltage-dependent Na^+^ and K^+^ currents [51]. Some cells were removed to access IHCs, but mostly, the pipette moved through the tissue under positive pressure. The extracellular solution was as follows (in mM): 155 NaCl, 5.8 KCl, 1.3 CaCl_2_, 0.9 MgCl_2_, 0.7 NaH_2_PO_4_, 5.6 d-glucose, and 10 Hepes buffer (pH 7.4). Two different pipette solutions were used. One contained (in mM): 150 KCl, 3.5 MgCl_2_, 0.1 CaCl_2_, 5 ethyleneglycol-bis(β-aminoethyl ether)-N,N,N′,N′- teraacetic acid (EGTA), 5 Hepes buffer, 2.5 Na_2_ATP, pH 7.2 (KCl-EGTA saline). In the other, EGTA was replaced with 10 mM bis(2-aminophenoxy)ethane-N,N,N′,N′-tetra-acetic acid (BAPTA) (KCl-BAPTA saline). The latter solution was used where indicated, in order to record the current through the ACh receptor in isolation from the coupled SK2 current (nAChR-only). In addition, in the latter condition, the SK blocker apamine (5 nM) was added to the recording solution.

Solutions containing ACh or high K^+^ were applied by a gravity-fed multichannel glass pipette (∼150 μm tip diameter), positioned about 300 μm from the recorded cell. All working solutions containing either ACh or elevated K^+^ or both, were made up in a saline containing low Ca^2+^ (0.5 mM) and no Mg^2+^ so as to optimize the experimental conditions for measuring currents flowing through the α9α10 receptors [38]. sIPSCs were recorded immediately after rupturing into the cell, in the extracellular saline containing 1.3 mM Ca^2+^ and no Mg^2+^. All experiments designed to record synaptic currents, either spontaneous or evoked with high K^+^, were done using an extracellular solution containing 1.3 mM Ca^2+^ and no Mg^2+^. sIPSCs were recorded immediately after rupturing the cell.

Glass pipettes, 1.2-mm I.D., had resistances of 5–8 MΩ. Currents in both IHCs and OHCs were recorded in the whole-cell patch-clamp mode with an Axopatch 200B amplifier, low-pass filtered at 2–10 kHz, and digitized at 5–20 kHz with a Digidata 1322A board (Molecular Devices). Recordings were made at room temperature (22–25 °C). Holding potentials were not corrected for liquid junction potentials or for the voltage drop across the uncompensated series resistance. IPSCs were analyzed with Minianalyis (Synaptosoft; Jaejin Software). IPSCs were identified using a search routine for event detection and confirmed by eye. The individual events of each cell were aligned and averaged using Minianalysis. Rise time was used as the criterion to align. For further analysis, Prism 4 (GraphPad Software) was used. The *τ*_rise_ and *τ*_decay_ were fit to a monoexponential.

Prolonged agonist application responses and concentration-response curves to ACh in IHCs were performed in the nAChR-only condition, normalized to the maximal agonist response, and iteratively fitted with the equation: *I/I_max_* = *A^n^*/(*A^n^* + EC_50_ *^n^*), where *I* is the peak inward current evoked by agonist at concentration *A*; *I_max_* is current evoked by the concentration of agonist eliciting a maximal response; EC_50_ is the concentration of agonist inducing half-maximal current response, and *n* is the Hill coefficient.

### Auditory brainstem responses and distortion product otoacoustic emissions.

Mice were anesthetized with xylazine (20 mg/kg i.p.) and ketamine (100 mg/kg i.p.). DPOAEs at 2f_1_–f_2_ were recorded with a custom acoustic assembly consisting of two electrostatic drivers (TDT EC-1; Tucker-Davis Technologies) to generate primary tones (f_1_ and f_2_ with f_2_/f_1_ = 1.2 and f_2_ level 10 dB < f_1_ level) and a Knowles miniature microphone (EK3103) to record ear-canal sound pressure. Stimuli were generated digitally, while resultant ear-canal sound pressure was amplified and digitally sampled at 4 μs (16-bit DAQ boards, NI 6052E; National Instruments). Fast Fourier Transforms were computed and averaged over five consecutive waveform traces, and 2f_1_–f_2_ DPOAE amplitude and surrounding noise floor were extracted. Iso-response curves were interpolated from plots of amplitude versus sound level, performed in 5-dB steps of f_1_ level. “Threshold” is defined as the f_1_ level required to produce a DPOAE with amplitude = 0 dB sound pressure level (SPL). For measurement of ABRs, needle electrodes were inserted at vertex and pinna, with a ground near the tail. ABRs were evoked with 5-ms tone pips (0.5-ms rise-fall, cos^2^ onset, at 35/s). The response was amplified (10,000×), filtered (100 Hz–3 kHz), and averaged with an A-D board in a LabVIEW-driven data-acquisition system. Sound level was raised in 5-dB steps from 10 dB below threshold to 80 dB SPL. At each level, 1,024 responses were averaged (with stimulus polarity alternated), using an “artifact reject” whereby response waveforms were discarded when peak-to-peak amplitude exceeded 15 μV. Upon visual inspection of stacked waveforms, “threshold” was defined as the lowest SPL level at which any wave could be detected, usually the level step just below that at which the response amplitude exceeded the noise floor (∼0.25 μV). For amplitude versus level functions, wave-I peak was identified by visual inspection at each sound level and the peak-to-peak amplitude computed.

### Olivocochlear assay.

Mice were anesthetized with urethane (1.20 g/kg i.p.) and xylazine (20 mg/kg i.p.). A posterior craniotomy and partial cerebellar aspiration were performed to expose the floor of the IVth ventricle. To stimulate the MOC bundle, shocks (monophasic pulses, 150-μs duration, 200/s) were applied through fine silver wires (0.4-mm spacing) placed along the midline, spanning the olivocochlear decussation. Shock threshold for facial twitches was determined, muscle paralysis induced with α-d-tubocurarine (1.25 mg/kg i.p.), and the animal connected to a respirator via a tracheal cannula. Shock levels were raised to 6 dB above twitch threshold. During the MOC suppression assay, f_2_ level was set to produce a DPOAE 10–15 dB or 20–25 dB greater than the noise floor. To measure MOC effects, repeated measures of baseline DPOAE amplitude were first obtained (*n* = 56), followed by a series of 70 contiguous periods in which DPOAE amplitudes were measured with simultaneous shocks to the MOC bundle and additional periods during which DPOAE measures continued after the termination of the shock train.

### Acoustic overexposure.

Animals were exposed free-field, in a small reverberant chamber. Acoustic trauma consisted of a 2-h exposure to an 8–16-kHz octave band noise presented at 100 dB SPL (for permanent injury) or a 15-min exposure to the same noise at 94 dB SPL (for temporary injury). For the higher level exposure, animals were anesthetized (ketamine and xylazine, exactly as for the ABR and DPOAE testing), because many mutant animals experienced audiogenic seizures as soon as the high-level noise was turned on. The exposure stimulus was generated by a custom white-noise source, filtered (Brickwall Filter with a 60-dB/octave slope), amplified (Crown power amplifier), and delivered (JBL compression driver) through an exponential horn fitted securely to a hole in the top of a reverberant box. Sound exposure levels were measured at four positions within each cage using a 0.25'' Bruel and Kjaer condenser microphone: sound pressure was found to vary by less than 0.5 dB across these measurement positions.

### Data analysis.

Statistical analyses of in vitro electrophysiology experiments were carried by the Student *t*-test in the case of pairwise comparisons or a one-way ANOVA followed by a Dunnet test for multiple comparisons. In the case of in vivo data, a two-way ANOVA was performed. A *p* < 0.05 was selected as the criterion for statistical significance. Mean values are quoted as means ± the standard error of the mean (S.E.M.).

### Materials.

ACh chloride, strychnine HCl, Na_2_ATP, BAPTA, and all other reagents were from Sigma Chemical. EGTA and Na_2_ATP were dissolved at the moment of preparing the intracellular solutions.

### Animal welfare.

All experimental protocols were carried out in accordance with the National Institutes of Health guide for the care and use of laboratory animals as well as Instituto de Investigaciones en Ingeniería Genética y Biología Molecular (INGEBI), Tufts University, and Massachusetts Eye and Ear Infirmary Institutional Animal Care and Use Committee (IACUC) guidelines, and best practice procedures.

## Supporting Information

Figure S1 Morphology of the Cochlear Duct Is Normal in Mutant Ears Gross histological assessment via plastic sections of osmium-stained *Chrna9^L9′T/L9′T^* cochleae showed no morphological changes when compared to *Chrna9^wt/wt^.* Sections through the middle turn are shown. Black and grey arrows in *Chrna9^wt/wt^* indicate OHCs and IHCs, respectively. Histological procedures were performed as described in Text S1.
(2.38 MB TIF)

Figure S2 Voltage-Dependent K^+^ Currents from IHCs and OHCs (A) Voltage-dependent currents obtained in P6–P10 IHCs by holding the cell at −80 mV and stepping the voltage to 80 mV in 10-mV increments (200-ms duration). Representative traces are shown for the three genotypes as well as for averaged steady state current-voltage curves (*Chrna9^wt/wt;^ n* = 10 animals, 13 cells; *Chrna9^wt/L9′T^*: *n* = 9 animals, 13 cells; and *Chrna9^L9′T/L9′T^*: *n* = 14 animals, 17 cells); (B) same as in (A), but for P10–P14 OHCs (*Chrna9^wt/wt;^ n* = 2 animals, 3 cells; and *Chrna9^L9′T/L9′T^*: *n* = 6 animals, 8 cells).
(225 KB TIF)

Figure S3 Dose-Response Curve for Strychnine Blockade of Cholinergic OC Effects on DPOAE Thresholds Each point shows data from different animals extracted from runs such as those shown in Figure 8 (f_2_ = 22.6 kHz). All data were collected 60 min after strychnine injection. Although thresholds in wild-type mice slightly rise with strychnine dosage, thresholds in knockin mice improve by as much as 8 dB.
(117 KB TIF)

Table S1 Analysis of Gene Expression by Quantitative PCR *Chrna9*: gene encoding the α9 nAChR subunit; *Chrna10*: gene encoding the α10 nAChR subunit; *Kcnn2*: gene encoding the SK2 channel; *Ryr1*: gene encoding the ryanodine receptor 1; *Ryr2*: gene encoding the ryanodine receptor 2; *Ryr3*: gene encoding the ryanodine receptor 3; *Kcnmb1*: gene encoding the calcium-activated potassium channel (BK) β-1subunit; *Cacna1d*: gene encoding the voltage-gated calcium channel alpha subunit CaV1.3; *Myo7a*: gene encoding myosin VIIa. C_Ts_: threshold cycle of sample; C_Tm_: threshold cycle of *Myo7a*. Results shown are the mean ± S.E.M of three experiments per group, each performed in triplicates. The protocol for quantitative PCR was as indicated in Text S2.
(88 KB DOC)

Text S1 Histology of the Cochlear Duct (24 KB DOC)

Text S2 Quantitative PCR (27 KB DOC)

## Abbreviations

ABR: auditory brainstem response
ACh: acetylcholine
DPOAE: distortion product otoacoustic emission
EC50: concentration that produces 50% of the maximal response
ES: embryonic stem
IHC: inner hair cell
IPSC: inhibitory postsynaptic current
L9′T: threonine for leucine mutation
i.p.: intraperitoneally
MOC: medial olivocochlear
nAChR: nicotinic acetylcholine receptor
OC: olivocochlear
OHC: outer hair cell
S.E.M.: standard error of the mean
sIPSC: spontaneous inhibitory postsynaptic current
