# Supplementary material for: A Point Mutation in the Hair Cell Nicotinic Cholinergic Receptor Prolongs Cochlear Inhibition and Enhances Noise Protection
Source: PLoS Biol. 2009 Jan 20;7(1):e1000018. doi: 10.1371/journal.pbio.1000018 (PMC2628405; doi:10.1371/journal.pbio.1000018)
Supplement: Supplementary file 1 [file pbio.1000018.sd001.doc]

**Text S1. Cochlear processing for histology**

Adult P42 mice were perfused intracardially with 2.5% glutaraldehyde and 1.5% paraformaldehyde in phosphate buffer for gross histological assessment via plastic sections of osmium-stained cochleae. Cochleae were decalcified in EDTA, osmicated and dehydrated in ethanol and propylene oxide, embedded in Araldite resins, and sectioned at 40μm on a Historange with a carbide steel knife. Sections were mounted on slides, coverslipped and analyzed under a light microscope.
