# Supplementary material for: A Point Mutation in the Hair Cell Nicotinic Cholinergic Receptor Prolongs Cochlear Inhibition and Enhances Noise Protection
Source: PLoS Biol. 2009 Jan 20;7(1):e1000018. doi: 10.1371/journal.pbio.1000018 (PMC2628405; doi:10.1371/journal.pbio.1000018)
Supplement: Supplementary file 2 [file pbio.1000018.sd002.doc]

**Text S2. Quantitative PCR**

Primers to selected genes of interest were purchased from Qiagen. Cochleas were collected and frozen in liquid nitrogen. During harvest and prior to freezing, the inner ear fossa bounded by the semi-circular canals was inspected to ensure that no cerebellum was inadvertently pulled off with the temporal bone. Cochleae from P42 old mice were frozen and stored in liquid nitrogen. Processing was performed as follows: 1) tissue was first weighed to assess the volume of Trizol (Invitrogen, Carlsbad, California) needed in subsequent steps; 2) tissue was ground in a TH-1 homogenizer (OMNI, Marietta, Gainsville) with Trizol; and 3) RNA was prepared according to the manufacturers instructions, with two minor modifications. A first spin was performed at 2500 xg to sediment the bone, and the supernatant was transferred to a fresh tube. Precipitation of the RNA was performed using 0.8M sodium citrate, 1.2M sodium chloride in addition to the isopropanol suggested in the manufacturer’s instructions. RNA concentration was assessed using a NanoDrop system, and integrity was assessed via electropherograms generated on an Agilent Technologies Bioanalyzer labchip (RNA 6000 Nano Assay mode).

qPCR analyses were performed using a one step RT-PCR procedure employing the QuantiTect SYBR Green RT-PCR kit (Qiagen), HotStarTaq DNA polymerase (Qiagen), and the QuantiTect primers of choice following manufacturer’s recommendations. Cycle-by-cycle data was collected as a SYBR Green fluorescence assay. PCR reactions were performed on a Strategene MX4000 instrument. Analysis of relative fold change in gene expression was calculated via the ΔΔC_T_ method [1] using myosin VIIa as a standard. Three independent experiments were performed, each one containing triplicates of pools of 4 or 6 cochleae. Amplification efficiency was similar for all primer sets used (not shown).

1. Schmittgen TD, Livak KJ (2008) Analyzing real-time PCR data by the comparative C(T) method. Nat Protoc 3: 1101-1108.
