# Supplementary material for: A Point Mutation in the Hair Cell Nicotinic Cholinergic Receptor Prolongs Cochlear Inhibition and Enhances Noise Protection
Source: PLoS Biol. 2009 Jan 20;7(1):e1000018. doi: 10.1371/journal.pbio.1000018 (PMC2628405; doi:10.1371/journal.pbio.1000018)
Supplement: Supplementary file 3 [file pbio.1000018.st001.doc]

| Gene  Subunit | Genotype | C_Ts_ | C_Tm_ | ΔC_T_ | ΔΔC_T_ | Expression level (2^-ΔΔCT^) (normalized to *Myo7a*) |
| --- | --- | --- | --- | --- | --- | --- |
| *Chrna9* | *Chrna9^L9T/L9T^* | 32.13±0.43 | 25.91±0.84 | 6.22 | -0,08 | 1.05 |
|  | *Chrna9^wt/wt^* | 32.43±0.55 | 26.14±1.23 | 6.29 |  |  |
| *Chrna10* | *Chrna9^L9T/L9T^* | 27.14±0.16 | 25.99±0.27 | 1.15 | 0.02 | 0.99 |
|  | *Chrna9^wt/wt^* | 27.38±0.55 | 26.25±0.51 | 1.13 |  |  |
| *Kcnn2* | *Chrna9^L9T/L9T^* | 26.08±0.30 | 25.99±0.27 | 0.09 | 0.34 | 0.79 |
|  | *Chrna9^wt/wt^* | 26.01±0.64 | 26.25±0.51 | -0.25 |  |  |
| *Ryr1* | *Chrna9^L9T/L9T^* | 22.36±0.24 | 25.99±0.27 | -3.63 | 0.15 | 0.90 |
|  | *Chrna9^wt/wt^* | 22.48±0.35 | 26.25±0.51 | -3.77 |  |  |
| *Ryr2* | *Chrna9^L9T/L9T^* | 24.83±0.39 | 26.28±0.03 | -1.45 | 0.30 | 0.81 |
|  | *Chrna9^wt/wt^* | 25.15±.079 | 26.90±0.17 | -1.75 |  |  |
| *Ryr3* | *Chrna9^L9T/L9T^* | 27.08±0.90 | 25.74±0.29 | 1.34 | 0.14 | 0.91 |
|  | *Chrna9^wt/wt^* | 27.01±0.42 | 25.81±0.56 | 1.20 |  |  |
| *Kcnmb1* | *Chrna9^L9T/L9T^* | 30.52±0.50 | 25.99±0.27 | 4.54 | 0.34 | 0.79 |
|  | *Chrna9^wt/wt^* | 30.46±0.64 | 26.25±0.51 | 4.20 |  |  |
| *Cacna1d* | *Chrna9^L9T/L9T^* | 28.03±0.87 | 26.02±0.28 | 2.01 | 0.51 | 0.70 |
|  | *Chrna9^wt/wt^* | 27.95±0.79 | 26.45±0.60 | 1.50 |  |  |

**Table S1: Analysis of gene expression by Quantitative PCR**
